# A Brief Review on Solvent-Free Synthesis of Zeolites

**DOI:** 10.3390/ma14040788

**Published:** 2021-02-07

**Authors:** Jinlin Mei, Aijun Duan, Xilong Wang

**Affiliations:** State Key Laboratory of Heavy Oil Processing, China University of Petroleum, Beijing 102249, China; meijinlinwh@163.com

**Keywords:** zeolites, solvent-free method, mechanism, applications

## Abstract

The traditional hydrothermal method to prepare zeolite will inevitably use a large amount of water as a solvent, which will lead to higher autogenous pressure, low efficiency, and wastewater pollution. The solvent-free method can be used to synthesize various types of zeolites by mechanical mixing, grinding, and heating of solid raw materials, which exhibits the apparent advantages of high yield, low pollution, and high efficiency. This review mainly introduces the development process of solvent-free synthesis, preparation of hierarchical zeolite, morphology control, synthesis mechanism and applications of solvent-free methods. It can be believed that solvent-free methods will become a research focus and have enormous industrial application potential.

## 1. Introduction

Zeolites, a kind of crystalline microporous material with outstanding hydrothermal stability, are one of the most important industrial inorganic solids [1,2,3,4], which have been widely applied in the field of ion exchange, adsorption, separation, medicine, optoelectronics and catalysis [5,6,7,8,9,10,11]. Zeolites can be obtained from natural ores or artificially synthesized. Generally, artificially synthesized zeolites have higher crystallinity and are widely used for ion exchangers and catalysts [12]. Hydrothermal synthesis has become a conventional method for zeolite preparation since Barrer and Milton proposed hydrothermal synthesis technology by simulating the natural conditions [13].

However, the hydrothermal method inevitably uses a lot of water as a solvent. Though water can be considered harmless, the excessive addition of water can cause many problems including the following: (1) filling too high with water will produce higher autogenous pressure, which will lead to equipment safety problems; (2) the space utilization efficiency of the autoclave is low, and the product yield is low; (3) the pollution from wastewater [14]. Therefore, synthesizing zeolites under anhydrous conditions reducing or removing solvents from the synthesis process is of great significance and conforms to the concept of green chemistry [15,16].

Pioneering research has challenged the necessity of water during zeolite synthesis. In 1990, Xu et al. [17] developed a new vapor-phase transport (VPT) method for the synthesis of zeolites, successfully avoiding the use of large amounts of water solvents during the crystallization process. In 1994, Althoff et al. [18] used ammonium fluoride instead of water vapor and proved that the presence of water during the crystallization process is not necessary.

Although the VPT method can solve some of the problems, it still necessary to use a lot of solvents in the process of preparing xerogels. In 2012, Xiao et al. [19] reported a solvent-free method of preparing zeolites without solvents by mechanical grinding and heating raw materials without adding additional water. In the solvent-free process, there is no evident free water or the use of other solvents instead of water. It does not really mean that there is no water in the system. This review mainly introduces the development process of solvent-free synthesis, preparation of hierarchical zeolite, morphology control, synthesis mechanism and applications of solvent-free methods.

## 2. Vapor-Phase Transport Technology

**Figure 1:**
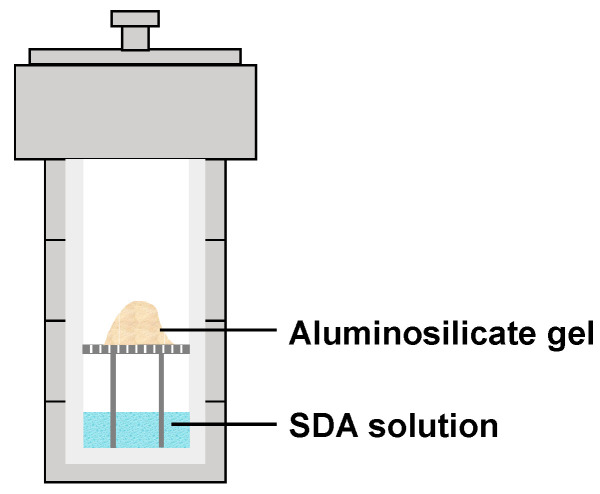
Synthesis of zeolites by vapor-phase transport (VPT) method.

In response to a series of problems caused by the excessive usage of water in the synthesis of zeolite, dry-gel conversion (DGC) and vapor-phase transport (VPT) techniques were developed [20]. The dry-gel sample was placed above the structure-directing agent (SDA) solution and reacted with water and amine vapors at a sealed autoclave under elevated temperature and autogenous pressure (Figure 1) [17,21]. ZSM-5 was successfully synthesized at 180–200 °C for 5–7 days. The mixed solution of water and amine was separated from the solid phase, which facilitated the recovery of the solution and could be reused to avoid wastewater treatment. Moreover, the cost of organic amines occupied a large part of the synthesis of zeolite, which reduced the consequent cost of the product.

To explore the necessity of water in the VPT method, Althoff et al. [18] treated the precursor at 650 °C for 24 h to form a dry powder. In the subsequent crystallization process, they used ammonium fluoride instead of directly using water vapor and gradually reduced the water content in the system ending in an absolutely dry mixture. They found that zeolite was still synthesized successfully in the case of extremely anhydrous conditions, indicating that water was not necessary for the crystallization process. They suggested that SiF_4_ was a mobile phase formed by the transformation of the amorphous precursor.

Kim et al. [22] and Matsukata et al. [23] improved relevant studies and proved that the gas phase transport technology could be applied to the synthesis of various zeolites, for example, MFI, FER, MOR and so on. Besides, the VPT method can expand the compositions of zeolite formation.

## 3. Solvent-Free Synthesis of Zeolite

The VPT technique addressed the problem of excessive usage of water in the crystallization process of zeolite. Through the success of VPT, it can be proposed that a solvent was not necessary for the crystallization process of zeolite. However, it is worth noting that an abundance of water was essential for the preparation of the gel. To address these problems, Xiao et al. [19] developed a solvent-free method based on mechanochemistry. Several of the most industrially important zeolites, with MFI, MOR, FAU, SOD, and *BEA frameworks, were successfully synthesized with this approach. The specific surface area, pore volume and pore size of the solvent-free ZSM-5 were 276 m^2^·g^−1^, 0.13 cm^3^·g^−1^ and 0.53 nm, respectively. Interestingly, zeolite seeds could replace SDA to direct the synthesis of Beta zeolite under solvent-free conditions, which could greatly reduce the use of SDA. Removing the use of expensive and toxic organic templates and excessive solvents can reduce the costs of zeolite synthesis and environmental pollution caused by calcined template agents and wastewater discharge, which would be the ultimate goal of synthesizing zeolite in an environmentally friendly and economical way.

Xiao et al. [24] conducted extended research on solvent-free synthesis combined with the template-free method with the assistance of NH_4_F. Eventually, silicalite-1, Beta, EU-1, and ZSM-22 zeolites were successfully synthesized. However, zeolite seeds were still necessary by this method. Thus, how to avoid using zeolite seeds should be the next research focus.

Gao et al. [25] successfully synthesized a high-crystallinity MOR zeolite in laboratory-scale conditions (650 g MOR was produced by 1 L autoclave) through the solvent-free method without an organic template. The specific surface area and pore volume of MOR zeolites synthesized at 473 K for 48 h were 392.1 m^2^·g^−1^ and 0.314 cm^3^·g^−1^, respectively, which are similar to those synthesized by conventional methods (397.9 m^2^·g^−1^ and 0.093 cm^3^·g^−1^). Though the pore volume was much larger than conventional MOR zeolites, the relative crystallinity was only 70%, which required further improvement. Notably, MFI structure appeared when SBA-15 was used as silica source, which indicated that it was possible to synthesize the MFI structure through a solvent-free and template-free method. Nada et al. [26,27] conducted related studies; they found that different zeolites can be synthesized by changing the ratio of raw materials. The higher Si content and lower Na content in the mixture were beneficial to ZSM-5 zeolites, while higher Na and Al contents were beneficial to mordenite, which was similar to the observations of synthesis. Currently, only a few zeolites can be synthesized without the use of templates and zeolite seeds by the solvent-free method.

In addition to the synthesis of aluminosilicates, the solvent-free method was extended to the preparation of aluminophosphates. Jin et al. [28] reported the use of the solvent-free method in the synthesis of silicoaluminophosphate, aluminophosphate, and heteroatom containing aluminophosphate zeolites. The specific surface area and pore volume of the solvent-free SAPO-34 were 459 m^2^·g^−1^ and 0.27 cm^3^·g^−1^, respectively. The solvent-free SAPO-34 had a conversion of close to 100%. The total yield was 88.9%, which was close to that of the SAPO-34 prepared by the traditional method (91.3%). Moreover, the obtained SAPO-34 exhibited the hierarchically porous structure which was beneficial for improving the selectivity of propylene and butylene and reducing ethylene selectivity.

It is worth noting that heteroatom can be conveniently incorporated into the framework of zeolites by solvent-free method, which has been commonly used in industry [29,30,31,32,33]. Generally, metal-exchanged zeolites were synthesized by performing multiple ion exchanges under hydrothermal conditions, which was complicated and also produced a lot of wastewater. Using the solvent-free method to synthesize heteroatom zeolites could overcome those shortcomings. Ma et al. [34] synthesized heteroatom zeolites in one pot through the solvent-free method by mechanical mixing, grinding and heating raw materials (aluminosilicate gel, templates, and metal-amine complexes). The ^27^Al MAS NMR spectrum of Cu-SSZ-13 has shown that there were no extra-framework aluminum species in the sample, which was in good agreement with the conventional hydrothermal method. Moreover, the yield of Cu-SSZ-13 was 98.1%, which was much higher than traditional ion exchange (55.6%) [35].

The solvent-free method could also be applied to metal-doped silicoaluminophosphate zeolites. Wang et al. [36] synthesized nanoscale Mg incorporated SAPO-34 by the solvent-free method. In this process, layered clay mineral, a cheap Mg-rich aluminosilicate, was used as Mg, Si and Al source. Besides, it also acted as a hard template to significantly reduce crystal size. The product had an Mg content of up to 6.65% and a small crystal size (50 nm), which was conducive to mass transfer. Zeolites with an Mg content of 3% exhibited a chloromethane conversion of almost 100% and a high light olefin selectivity of 88.1%.

## 4. Formation of Hierarchical Pore Structure

Zeolites have received extensive attention due to its easily adjustable physicochemical properties. However, the diffusion limitation of micropores will constrain the mass transfer of the reactants and products, thereby reducing the kinetics of the reaction. Therefore, the hierarchically porous structure is necessary for macromolecular reactions. Commonly, the synthesis method of hierarchically porous zeolites is classified into bottom-up and top-down methods [37,38,39]. In the hydrothermal synthesis process, different methods that utilize hard-template or soft-template are categorized as bottom-up approaches, while those by treating zeolites by dealumination and desilication are classified as top-down methods [39]. Herein, the concept is still used in the solvent-free method.

### 4.1. Bottom-Up Strategy

Jin et al. [40] synthesized hierarchically porous aluminophosphate-based zeolites through the solvent-free method. In the synthesis of zeolites with AEL structure (APO-11), di-n-propylamine phosphate (DPA·H_3_PO_4_) and boehmite were ground at room temperature and heated at 200 °C. After calcination at 600 °C for 4 h, the relative pressure of the sample increased sharply at 10^−6^–0.01, which could be attributed to the filling of micropores. Meanwhile, a hysteresis loop can be observed at a relative pressure of 0.45–0.95, suggesting the presence of mesoporosity and macroporosity in the sample. DPA·H_3_PO_4_ was served as an organic template and phosphorus source, and avoided the volatilization of amines during synthesis. Sheng et al. [41] found that a large amount of gaseous di-n-propylamine (DPA) molecules could be quickly released from DPA·H_3_PO_4_ during the crystallization process, which could be served as “porogens” for the formation of mesoporosity.

**Figure 2:**
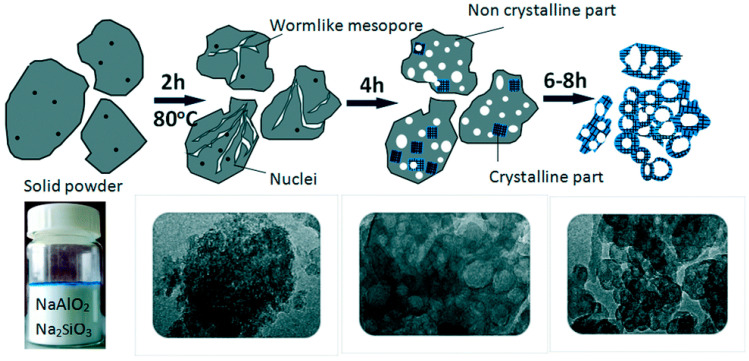
Schematic process of the growth mechanism for hierarchically porous SOD via solvent-free method. Reproduced from Ref. [42] with permission from The Royal Society of Chemistry.

Zeng et al. [42] synthesized hierarchically porous SOD zeolites by the solvent-free method with organosilane as a mesopore-generating agent. Figure 2 illuminated the schematic process of the growth mechanism for hierarchically porous SOD via the solvent-free method. Before crystallization after grinding, the samples formed an amorphous solid. After crystallization for 2 h, worm-like mesoporous channels resulting from amorphous aggregates could be observed. After crystallization for 4 h, voids or cavities were detected, which would result in the formation of cell-like SOD zeolite. The synthesized SOD zeolites exhibited small mesopores (4.6 nm) owing to organosilane surfactant and meso-macropores (20–55 nm) attributed to interparticle voids.

In addition to the soft templates mentioned above, hard templates were also feasible. Ren et al. [19] found that after the addition of CaCO_3_ in the solid raw materials as a hard template followed by acid treatment, ZSM-5 crystals possessed macropores about 100 nm. Moreover, Li et al. [43] used graphene oxide (GO) sheets as a hard template to prepare hierarchically porous ZSM-5. After removing the template, the sizes of the micropores and mesopores were about 0.6 nm and 2–60 nm, respectively.

### 4.2. Up–Down Strategy

Organic compounds were usually served as a secondary template or porogen to assist the formation of mesopores or macropores [44] via a bottom-up strategy that was not environmentally friendly and economical. The up–down strategy could eliminate the use of mesoporogen.

Hierarchically porous HY zeolites were prepared by Nichterwitz et al. [45] using carbochlorination in solvent-free conditions. HY zeolites were ground with furfuryl alcohol (FA) and ethanol (EtOH) followed by temperature-programmed carbonized and carbochlorination. As the chlorination temperature increased, the total pore volume and mesopore volume increased, while the micropore volume decreased. Meanwhile, the pore size became larger. The apertures of samples chloridized at 673 K and 773 K were 4 and 7 nm, respectively. For the samples chloridized at 873 K, the pore size distributed from 6 to 16 nm. While chloridized at 973 and 1073 K, samples showed two main pore sizes of 10 and 16 nm. Carbon content in composite, chlorination time and Si/Al ratio might also affect the physicochemical properties of zeolites. Carbochlorination can double the pore volume, while the surface area and crystallinity of the material can be maintained under optimal conditions.

**Figure 3:**
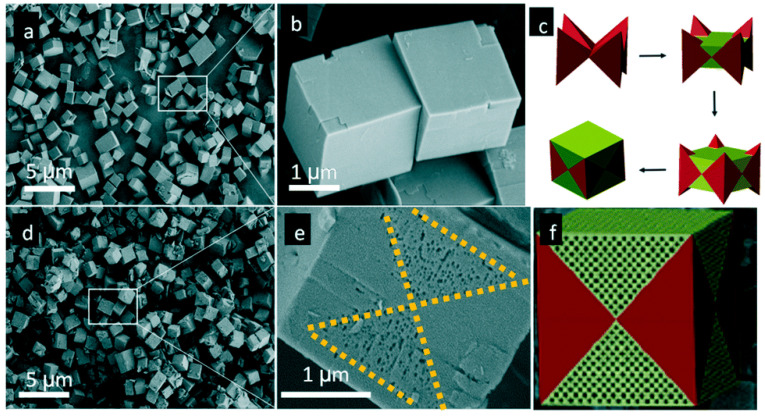
SEM images of SAPO-34 (**a**,**b**) before and (**d**,**e**) after solid oxalic acid treatment, (**c**) schematic drawing of the growth of SAPO-34, and (**f**) the drawing of the morphology of treated SAPO-34. Reproduced from Ref. [46] with permission from The Royal Society of Chemistry.

Though carbochlorination was effective, its conditions were relatively severe (around 800 °C). Dealumination or desilication by acids or bases were adopted alternatively. Hierarchically porous SAPO-34 zeolites were synthesized by grinding SAPO-34 with solid oxalic acid followed by heating at 373 K for 6 h [46]. Interestingly, the surface of the product presented a clear pattern of butterfly pores with a pore size of 60–100 nm. The formation of butterfly pattern SAPO-34 was related to the synthesis process of the cubic SAPO-34 and the following etching process. In the early stages of the synthesis of cubic SAPO-34, crystals had preferentially grown in a specific direction, thus forming a specific crystal morphology, which consisted of eight cone parts and a void around the center. Then, the void was filled along with crystal growth to form a perfect cube (Figure 3c). However, the energy of subsequently filled parts was unstable. Therefore, it was preferable to etch them after treatment with solid oxalic acid to form a layered structure with a butterfly pattern.

Hierarchically porous SAPO-11 zeolites were also prepared by this method [47]. It was discovered that high crystallinity and uniform morphology were retained after etching by solid oxalic acid. In addition, more (002) crystal planes were exposed meaning more acidic sites could be provided. The results of N_2_ adsorption–desorption showed that though the mesoporous structure (4 nm) was dominant, all samples contained microporous (0.5 nm) and mesoporous structures.

## 5. Synthesis of Zeolites with Different Morphologies

It was reported that the morphologies of zeolites might improve mass transfer, because the physicochemical properties of zeolites could be strongly affected by their morphologies [48,49,50].

**Figure 4:**
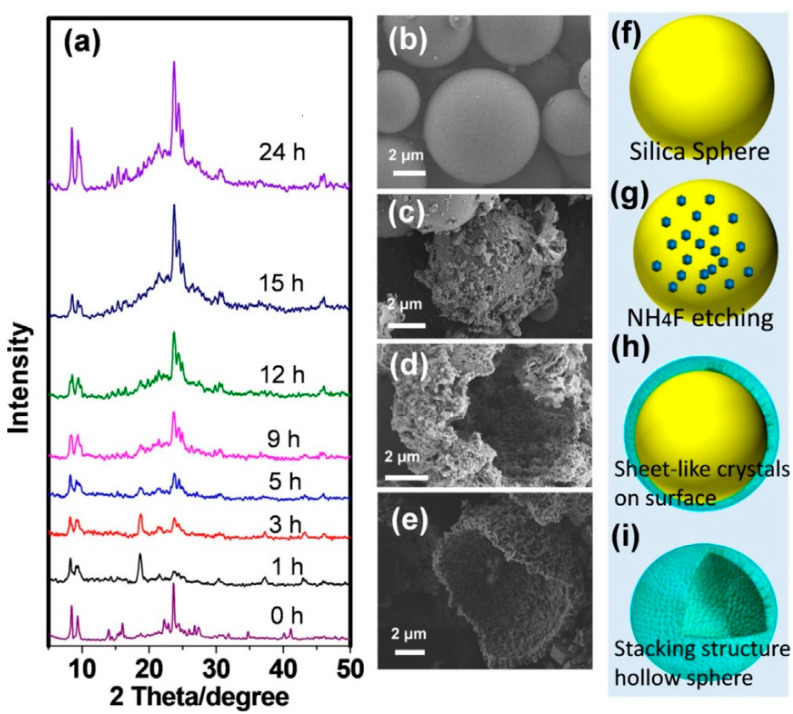
(**a**) In-situ XRD patterns, (**b**–**e**) SEM images, and (**f**–**i**) corresponding illustration of hollow ZSM-5 shell at different crystallization times. Reproduced from Ref. [51] with permission from 2019 Wiley-VCH Verlag GmbH & Co. KGaA, Weinheim.

Wu et al. [51] synthesized a hollow zeolite shell assembled by ZSM-5 with a short b-axis by the solvent-free method. SiO_2_ spheres were used as silica source, with prefabricated ZSM-5 and NH_4_F as Al source and mineralizer, respectively. In-situ XRD analysis and SEM were performed to analyze the morphological and structural evolutions. After crystallizing for 1 h, the peaks of MFI significantly weakened, indicating that prefabricated ZSM-5 was broken down into small segments. Meanwhile, the peak at 18.68° was associated with (NH_4_)_2_SiF_6_, inferring that NH_4_F reacted with silica from the substrate and prefabricated ZSM-5 which roughen the surface of the silica ball (Figure 4b,c). No (NH_4_)_3_AlF_6_ peaks appeared in all samples, indicating that the confined Al source prevented coordination between Al and the fluoride species giving a well-preserved acidic framework Al in the final products. After crystallizing for 2–4 h, SiF_6_^2−^ species were gradually condensed to Si(OSi)_4_ in the initial stage of MFI crystallization (Figure 4d). As the crystallization was prolonged, SiF_6_^2−^ species were completely converted into ZSM-5 (Figure 4e). During crystallization, silica was migrated to the surface leading to the formation of the hollow structure.

In hydrothermal synthesis, additives were often used to adjust crystal morphology [52], which were also used in the solvent-free method. Zhang et al. [53] synthesized silicalite-1 zeolites by the solvent-free method with the assistance of NH_4_F. It was discovered that the addition of NaOH (NaOH/SiO_2_ = 0.09) could reduce the crystal sizes and the samples were in a morphology of coffin which had a long c-axis. As the content of NaOH increased (NaOH/SiO_2_ = 0.19), the morphology of crystals became spherical.

Chen et al. [54] synthesized SAPO-5 zeolites with plate-like morphology with cetyltrimethylammonium bromide (CTAB) as a surfactant through the solvent-free method. It was discovered that CTAB strongly suppressed the growth of SAPO-5 crystal in the (002) direction. The morphology of the product was spherical in the absence of CTAB. When the amount of CTAB added increased, the morphology of the zeolite gradually changed from a spherical shape to a discus-like shape, and finally to a sheet shape. Different from the formation of self-assembly micelles in the solvent, CTAB could selectively adsorb on certain surfaces of the SAPO-5, thereby inhibiting the growth in the (002) direction.

Yu et al. [55] synthesized well-crystallized hollow fibers composed of c-axis-oriented ZSM-5 by the solvent-free method with quartz fiber as silicon source and NH_4_HCO_3_ as additives. During the process of crystallization, the silicon source continuously migrated to the surface of the quartz fiber and crystallized on the surface to form ZSM-5, giving a hollow morphology. Moreover, the length of the c-axis of ZSM-5 was adjustable by controlling the amount of NH_4_HCO_3_. NH_3_ decomposition by NH_4_HCO_3_ could inhibit the growth of (010) surface. Liu et al. [56] also synthesized c-axis-oriented ZSM-5 by the solvent-free method in the presence of urea. It was discovered that NH_3_ decomposition by urea suppressed the growth of b-axis orientation and developed the growth in the c-axis direction.

Graphene oxide (GO) sheets were also used as an additive to synthesize c-axis-oriented ZSM-5 by the solvent-free method [43]. It was discovered that GO sheet adsorbed more stably on (010) and (100) planes than on (101) planes leading to the growth restriction of the (010) and (100) facets. So, ZSM-5 crystals had an elongated shape along the c-axis, which was analogous to the conclusion of hydrothermal synthesis [57]. Moreover, GO could prevent the aggregation of the ZSM-5 crystals during the synthesis. Furthermore, the addition of GO sheets led to the formation of hierarchical pore structures, which is conducive to mass transfer and product selectivity [58].

## 6. Mechanism on Zeolite Synthesis

**Figure 5:**
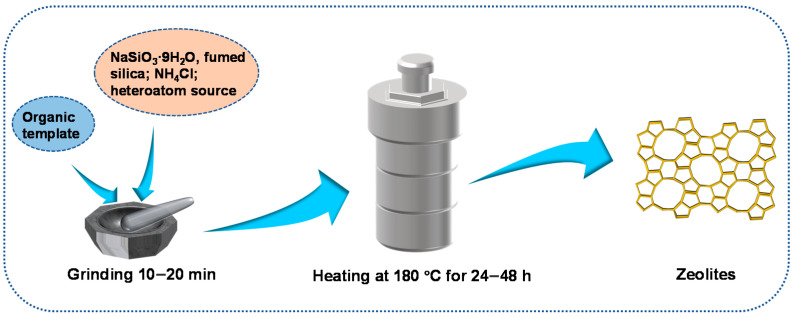
Synthesis of zeolites through the solvent-free method.

Xiao et al. [19] first reported the solvent-free method in 2012. Typically, the solid raw materials (NaSiO_3_·9H_2_O, fumed silica, NH_4_Cl, organic SDA and heteroatom source such as Al and Fe) were ground for 10–20 min without the addition of any water. Then the mixture was heated in an autoclave at 453 K for 24–72 h. Figure 5 is a schematic diagram of the solvent-free method.

They found that the sample after grinding showed diffraction peaks of NaCl, though NaCl was not one of the raw materials. It was believed that NaCl was obtained by the reaction between Na_2_SiO_3_ and NH_4_Cl, which was consistent with the research of Nada [27]. Ammonia, metasilicic acid and water were also generated in the process of grinding. Simultaneously, the trace amount of water from the hydrated raw material acted as a medium that diffused and intimately mixed Si, Al and O ions, promoting the growth of ZSM-5 crystals. Xiao et al. [19] found that that the ultraviolet Raman signal attributed to the template was significantly weakened after heating at 453 K for 2 h, indicating that the template was highly dispersed on the amorphous carrier. Moreover, the amorphous intermediate sample heating for 2 h exhibited a pore size of 0.8 nm, while there was no porosity in the intermediate materials at a similar stage in hydrothermal synthesis.

It was worth mentioning that when all raw materials do not contain crystal water, ZSM-5 cannot be obtained by solvent-free methods [59]. Trace water was a key factor for zeolite crystallization in the solvent-free route, which was similar to the role of “catalyst” that promoted depolymerization and condensation of silica species. To prove this point of view, solvent-free zeolite synthesis was performed from anhydrous solid raw materials with NH_4_F as a mineralizer, and a zeolite product with high crystallinity was obtained [24]. At the beginning of the synthesis process, SiF_6_^2−^ species produced by the reaction of silica and NH_4_F were observed. After crystallization for 2.5 h, SiF_6_^2−^ species were condensed to Si(OSi)_4_, and F^−^ was released. It was suggested that F^−^ could serve as a “catalyst” for depolymerization and condensation of silica species, similar to water.

**Figure 6:**
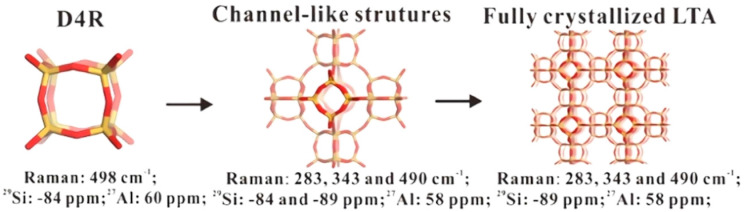
The proposed route for the synthesis of zeolite A in the absence of solvent. Reproduced from Ref. [60] with permission from 2016 Elsevier Inc.

The crystallization process of the ground mixture was demonstrated by NaA zeolite [60]. The result of UV-Raman characterization demonstrated that after 24 h of aging at room temperature, the ground sample showed a weak band at 498 cm^−1^, which was related to the four-membered rings (4R). It was consistent with the peak position of the sample after crystallization at 80 °C for 2 h. However, the as-ground sample did not exhibit the same band. This indicated that the raw materials could interact with each other at room temperature after grinding, and spontaneously form a four-membered rings (4R) structure. With the increase in crystallization time, in addition to 4R, six-membered rings (6R) and eight-membered rings (8R) appeared, and the intensities of these signals increased. The results of ^27^Al MAS NMR, ^29^Si NMR and ^27^Al 2D MQMAS NMR further showed that 4R in the parent mixture were double four-membered rings (D4R) rather than a single four-membered ring (S4R). After 4 h of crystallization, all D4R units had been converted into LTA structure. It can be concluded that hydrated silica and sodium aluminate would interact with each other spontaneously, leading to the formation of D4R, which could be further assembled or rearranged into NaA crystals in the absence of water solvent, as shown in Figure 6.

## 7. Applications of Solvent-Free Zeolite

Although the solvent-free method was a convenient method to synthesize zeolites, the performance of synthesized zeolite was unknown, which needed to be tested in practice. Wang et al. [61] successfully synthesized CHA zeolite SSZ-13 with N,N,N-dimethylethylcyclohexylammonium bromide as a template by the solvent-free method. The specific surface area and pore volume of the product were 584 m^2^·g^−1^ and 0.27 cm^3^·g^−1^, respectively, which was close to those of SSZ-13 prepared by a hydrothermal route. The solvent-free SSZ-13 was used in the methanol-to-olefins (MTO) reaction at 623 K. It was found that the conversion rate of methanol was close to 100%, and the selectivity of propylene was about 35%, which was similar to SSZ-13 synthesized by a hydrothermal method.

Liu et al. [62] synthesized a hierarchical ZSM-5 zeolite with high crystallinity, adjustable Si/Al ratio for methanol to gasoline (MTG) conversion by the solvent-free method [63]. Activated carbon was blended with other raw materials and ground for 20 s to form the hierarchical pore structure. Si/Al ratio was facilely adjusted from 20 to 100 by controlling the amount of NaAlO_2_ added. Solvent-free ZSM-5 exhibits excellent MTG performance, with a methanol conversion rate close to 100%, C5^+^ selectivity higher than 60% and a lifetime of 350 h.

Zhang et al. [63] also synthesized ZSM-5 by a solvent-free method applied in MTO reaction and compared it with ZSM-5 prepared by the traditional hydrothermal method. The characteristic diffraction peaks of ZSM-5 appeared when the crystallization time reached 3 h. When it reached 6 h, the crystallinity was similar to the traditional ZSM-5. When the crystallization time was 30 h and the Si/Al ratio of raw materials was 150 under the solvent-free conditions, the selectivity of the catalyst to propylene was as high as 50%, and the catalyst life was as long as 9 h, which were much better than those of the traditional ZSM-5 zeolites with propylene selectivity of 38.9% and catalyst life of 3 h. Moreover, the presence of mesoporous was confirmed by high-resolution TEM, which was favorable for the mass transfer. More interestingly, these mesopore size distributions were adjustable by controlling the crystallization time.

## 8. Conclusions and Perspective

Compared with the traditional hydrothermal method, the solvent-free synthesis method displays the following prominent advantages [19]. (1) High yields—a large amount of solvent used in traditional hydrothermal synthesis will dissolve partial nutrients such as silicate or aluminate, reducing the yield of the product. (2) High single-autoclave yield—the solvent occupies most of the space in the autoclaves during the hydrothermal synthesis process, otherwise, the autoclave can hold more solid raw materials through the solvent-free method; as such, the single-autoclave yield is greatly increased. (3) Fewer pollutants—solvents are not used in solvent-free methods, reducing wastewater emissions efficiently. (4) Low crystallization pressure—since solvents are not adopted in the solvent-free method, the autogenous pressure generated at high temperatures is relatively lower, which makes the requirements for equipment moderate. (5) High space-time yields—the long-term aging process does not exist in the solvent-free method, so the space-time yield is greatly increased. Table 1 lists the zeolites synthesized by the solvent-free method, including silica, aluminosilicate, and aluminophosphate zeolites.

In view of the above factors, the solvent-free route will be of great significance to the industrial production of zeolites in the future. Combined with the template-free method, the cost of the solvent-free method will also be reduced, making it more competitive than hydrothermal synthesis. However, in the solvent-free process, the template-free method can only be used to synthesize a few special zeolites, and most zeolites still require SDA. Although the use of zeolite seeds can expand the synthesis range of template-free and solvent-free methods, the preparation of seed crystals is still a relatively complicated step. Hence, how to synthesize zeolite solvent-free without using templating agents and zeolite seeds is the focus of future research.

In addition, insights into the mechanism of the solvent-free synthesis of zeolite are still necessary, especially regarding the interaction between SDA and framework. The current research results show that the primary and secondary structural units play a vital role in the crystallization process of zeolite, but the interaction between the template and the zeolite is not well understood. Different from the hydrothermal method, there is microporosity in the amorphous intermediate materials by solvent-free method indicating a distinguishable mechanism on the crystallization of zeolites. There is still a lack of appropriate theories to explain how SDA directs amorphous silica to a specific zeolite structure.

In summary, the solvent-free method is a simplified, efficient, high-yield, and low-cost methodology for the preparation of zeolites but is not limited to zeolites. A small amount of water was a critical parameter for zeolite formation. By combining the advantages of solvent-free and template-free synthesis, a large-scale application in zeolite synthesis would have great potential in the industry.

## Figures and Tables

**Table 1:** Zeolites synthesized by the solvent-free method.

Zeolite	IZA Code	Dimension	Crystal System	Space Group	Reference
ZSM-5	MFI	3	orthorhombic	P n m a	[16,19,24,43,51,64,65,66]
silicalite-1	MFI	3	orthorhombic	P n m a	[19,53]
Beta	*BEA	3	tetragonal	P 4_1_ 2 2	[16,24,66]
Y	FAU	3	cubic	F d $\bar{3}$ m	[19]
ZSM-39	MTN	0	cubic	F d $\bar{3}$ m	[19]
EU-1	EUO	1	orthorhombic	C m m e	[24]
ZSM-22	TON	1	orthorhombic	C m c m	[24]
Mordenite	MOR	2	orthorhombic	C m c m	[25,66,67]
TS-1	MFI	3	orthorhombic	P n m a	[68]
sodalite	SOD	0	cubic	I m $\bar{3}$ m	[19,42,69]
A	LTA	3	cubic	P m $\bar{3}$ m	[60]
SAPO-34	CHA	3	trigonal	R $\bar{3}$ m	[28]
SAPO-43	SOD	0	cubic	I m $\bar{3}$ m	[28]
SAPO-20	GIS	3	tetragonal	I 4_1_/a m d	[28]
SAPO-11	AEL	1	orthorhombic	I m m a	[28,40,70]
SAPO-5	AFL	1	hexagonal	P 6/m c c	[40,54]
SSZ-13	CHA	3	trigonal	R $\bar{3}$ m	[61,71]
ITQ-12	ITW	2	monoclinic	C1 2/m 1	[72]
ITQ-13	ITH		orthorhombic	A m m 2	[72]
ITQ-17	BEC	3	tetragonal	P 4_2_/m m c	[72]
Cancrinite	CAN	1	hexagonal	P 6_3_/m m c	[73]
Na-EMT	EMT	3	hexagonal	P 6_3_/m m c	[74]
SSZ-39	AEI	3	orthorhombic	C m c m	[75]

## Data Availability

Data sharing is not applicable to this article.

## Nomenclature

^27^Al 2D MQMAS NMR	^27^Al two-dimensional multiple-quantum magic-angle spinning nuclear magnetic resonance
^27^Al MAS NMR	^27^Al magic-angle spinning nuclear magnetic resonance
^29^Si NMR	^29^Si nuclear magnetic resonance
4R	Four-membered rings
6R	Six-membered rings
8R	Eight-membered rings
Al	Aluminum
C5^+^	Gasoline-range hydrocarbons
CTAB	Cetyltrimethylammonium bromide
DGC	Dry-gel conversion
DPA	Di-n-propylamine
DPA·H_3_PO_4_	Di-n-propylamine phosphate
EtOH	Ethanol
FA	Furfuryl alcohol
Fe	Iron
GO	Graphene oxide
IZA	International zeolite association
Mg	Magnesium
MTG	Methanol to gasoline
MTO	Methanol to olefins
Na_2_SiO_3_	Sodium silicate
NaAlO_2_	Sodium aluminate
NaCl	Sodium chloride
NaOH	Sodium hydroxide
Na_2_SiO_3_·9H_2_O	Sodium metasilicate nonahydrate
NH_3_	Ammonia
NH_4_Cl	Ammonium chloride
NH_4_F	Ammonium fluoride
NH_4_HCO_3_	Ammonium bicarbonate
(NH_4_)_2_SiF_6_	Ammonium silicofluoride
O	Oxygen
SDA	Structure-directing agent
SEM	Scanning electron microscope
Si	Silicon
SiO_2_	Silica
TEM	Transmission electron microscope
VPT	Vapor-phase transport
XRD	X-ray diffraction
